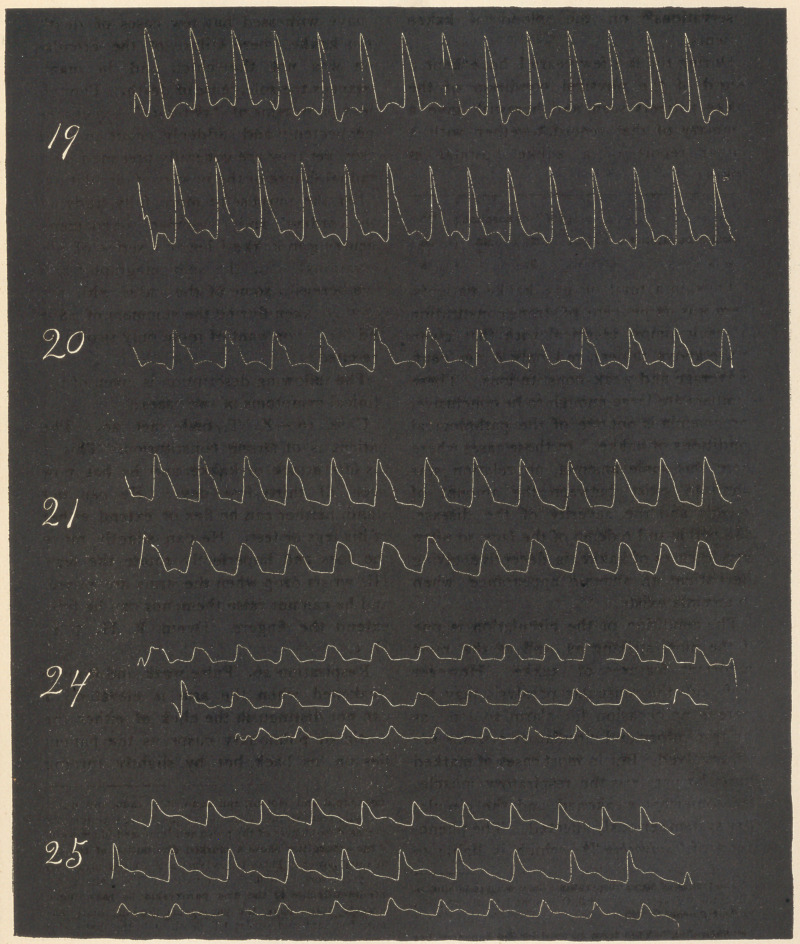# Japanese “Kakke”

**Published:** 1888-04

**Authors:** Wallace Taylor

**Affiliations:** Osaka, Japan


### STUDIES IN JAPANESE "KAKKE," OR BERI-BERI.

BY WALLACE TAYLOR, M. D.,
OF OSAKA, JAPAN.

*History.*—The most competent writers believe that a disease corresponding to that now known in Japan as kakke existed in China as early as the year 200, B. C. The term signifies disease of the legs.

The accounts given of this disease state that it appeared in the province of Tsin, during the Tsin dynasty, which existed from 267 to 317, A. D.; and that from thence it gradually spread over the country to the east and south of the river. The ancient province of Tsin included the southern half of Shansi and the north-west portion of Honan provinces.

It would appear that "kakke" was first known in this section of China, and gradually spread over the alluvial plain east along the Hoang Ho River, and then gradually became disseminated over a large portion of the country. But spreading over the country may mean no more than that this disease was then first recognized. In the crude state of medical knowledge of that time, the disease may have prevailed ages before unrecognized. Recent Chinese medical literature is silent in regard to this disease, hence it has been thought to have disappeared from the country. European physicians, practicing in China, have failed to recognize this disease till recently. Within the last two years Drs. Lambeth and Park of Tsuchow, and Dr. Boone of Shanghai, have met a number of cases. These gentlemen are of the opinion that there are many cases of beri-beri among the Chinese. The disease has also been recognized at Fatshan, near Canton, and among the Chinese in the Hong-Kong city hospital.

The earliest account of this disease in Japan is found in the political history of the country. As early as A. D. 809, the historical collections of "*Nihon Koeki*" record a letter of resignation of an officer to his superior in Kiyoto, assigning as the reason of his resignation that every year he suffered from "*Ashi ke yamai*" (disease of the legs), and was therefore unable to perform the duties of his office. The earliest account of this disease in Japanese medical literature dates back to about A. D. 880. Most of the earlier medical writers drew their description of this disease largely from Chinese medical works, but state that the disease was found in Japan. The earliest account of this disease in Japanese medical literature, bearing evidence of originality, dates back about 165 years. Since that date the references to the disease become more and more frequent, and show a better understanding of its nature. There is evidence conclusive to show, that within recent years it has been gradually extending to districts where it had hitherto been unknown.

*Pathology.*—The statements made in regard to the condition of the blood in beri-beri have been contradictory. Most authors state that anæmia is one of the causes of this disease, or if not a predisposing cause, an essential accompanying element, and also that there is an increase in the number of white corpuscles in the blood. Dr. Simmons was the first to contradict this prevailing opinion. The peculiar pallor which most generally accompanies this disease has undoubtedly led to this opinion. But this appearance is deceptive; it is not due to an impoverished condition of the blood, but to other causes.

Clinical experience had led me, several years ago, to the conclusion that anæmia was not a predisposing cause, and if found

19
20
21
24
25

complicating cases of kakke its association was accidental; and, to fully settle the question for myself, I began a series of observations* on the blood of kakee patients.

During the last few years I have kept a record of the physical condition of the kakke patients seen, and herewith give a summary of that record, together with a similar report of a kakke hospital in Tokio:

| | Taylor. | Kakke hospital. | Sum. |
|---|---|---|---|
| Of strong constitution ..... | 323 | 593 | 916 |
| " average " .......... | 15 | 28 | 42 |
| " weak " .......... | 9 | 6 | 15 |

Thus, in a total of 973 kakke patients, there was 94 per cent. of strong constitution (a result almost identical with that given in the above tables) and only 6 per cent. of average and weak constitutions. These numbers are large enough to be conclusive, and anæmia is not one of the pathological conditions of kakke. In those cases where there was some anæmia, no relation was found to exist between the amount of anæmia and the severity of the disease. The pallor and œdema of the face, so often seen in cases of kakke, is deceptive, giving the patient an anæmic appearance when no anæmia exists.

The condition of the circulation is one of the most striking as well as the most important features of kakke. However profound the muscular paralysis may be, there is no occasion for alarm so long as the respiratory and circulatory systems are not involved. But in most cases of marked muscular paralysis the respiratory muscles are somewhat weakened, and the circulatory system seriously affected. The phenomena of "*shiyoshin*"†—which is liable to suddenly occur at any time in any case—is chiefly due to failure of the circulation and respiration, especially the former. I have witnessed but few cases of death from kakke where failure of the circulation was not the chief, and in many instances the sole, cause of death. Though these paroxysms of "*shiyoshin*" sometimes unexpectedly and suddenly occur in mild cases, yet they are generally preceded by a gradual failure of the powers of circulation.

For the purpose of more fully studying the cardiac and vascular phenomena occurring in kakke I began a series of observations‡ with the sphygmograph,§ and give herewith some of the cases with the tracings taken during the summers of 1883 and '84. For want of room only two cases are cited:

The following description is given of the clinical symptoms of two cases:

CASE 16.—K. T., male, aet. 49. The patient is of strong constitution. This is his fifth attack of kakke, and he has now been ill thirty-five days. He can not stand, neither can he flex or extend either of his legs or feet. He can slightly move the toes and imperfectly rotate the legs. His wrists drop when the arms are raised, and he can not raise them, nor can he fully extend the fingers. Dynm. R. H. 5, L. H. 5.

Respiration 26. Pulse weak, and is more weakened when the arm is elevated. I can not distinguish the click of either the aortic or pulmonary cusps, as the patient lies on his back, but by slightly turning

* The results of these observations have been published in full in the "*Tokeizasshi*," 1883, Osaka, and in the "*Tokei Medical Journal*," 1884.

† "*Shiyoshin*."—This term as used by the Japanese has no pathological signification; they simply mean by it a metastasis of kakke to the chest. The paroxysms of "*Shiyoshin*" bear some resemblance to an acute attack of *Angina pectoris*. There is great distress in the chest, accompanied by marked dyspnœa, and failure of the powers of circulation. The action of the heart is sometimes rapid and violent, and again it is calm and quiet. But whatever be the character of the heart's action, the increased feebleness of the pulse and increased blueness of of the extremities show a marked diminution of the circulatory power. These paroxysms are almost invariably the precursors of a fatal termination. The patient not infrequently dies in the first paroxysms; he may linger, however, a few days, the paroxysms growing more and more frequent and severe till death ends the distressing scene.

‡ A full report of these observations are published in the "*Tokei Medical Journal*," 1885-86.

§ The tracings here given were taken with Marcy's Sphygmograph, improved by Mahomed; and the rate of travel by the slide bearing the card was 11 to 12 cm. in 10 seconds.

him over on his left side these valve sounds can be faintly heard. He has some distress in the chest, and suffers from palpitation of the heart.

Seven days later the patient is gradually losing ground. The hands and feet are cold and slightly purple. They look and feel much as the extremities of a person in collapse from cholera, except that the cuticle still retains its elasticity. The voice has grown very weak, and the patient is suffering from marked dyspnœa. The diaphragm is partially paralyzed, and respiration is largely carried on by the respiratory muscles of the chest.

But the following night slight "*shiyoshin*" developed, and the patient died.

CASE 19.—Y. C., male, aet. 19. The patient is of strong constitution. This was a mild case, and had been ill some thirty days. He was attending the hospital as an out-patient, continued his work, and did not appear much discommoded by his slight muscular paralysis. He had not reported himself for some ten days. He said he had been gradually growing worse for the last three days. Last night he was taken with pain in the bowels and distress in the chest. This gradually grew worse, and this morning he had several very copious movements of the bowels. He became faint and slight "*shiyoshin*" developed, when I was sent for. The paroxysm of "*shiyoshin*" had passed off before I reached the patient. The pulse was weak, there was marked pallor, the extremities were cold, and the patient was in a state of partial collapse.

Cases are frequently met with where the first prominent symptoms of kakke developed just as this did, with diarrhœa and distress in the chest. Such cases of "*shiyoshin*" are occasionally met, but diarrhœa does not generally accompany kakke; obstinate constipation is much more frequently met with.

Condition of blood: A large number of kakke micro-organisms were found in this patient's blood.

There was some œdema. The patient was at once given digitalis, strichnia, and whisky, and put upon an active course of cathartics and diuretics. The patient at once began to improve.

*Summary.*—There was no organic disease of the heart or arteries in any of the cases observed, and no functional cardiac derangement, except that due to the disease.

There are three characteristics in all kakke pulse tracings, viz.: (*a*) The very sudden and high upstroke of the ventricular systole. (*b*) The precipitous descent from the apex of the purcussion wave. (*c*) Dicrotism.

*The first deviation of the circulatory system in kakke from the normal is one of cardiac excitement.* In the early stages of kakke, and in mild cases, the sudden and tall upstroke of the percussion wave points to a condition of *cardiac excitement.* The first complaints that kakee patients made along with that of anæsthsia and œdema of the legs is (*ikidoshii*) of cardiac dyspnœa.

The low condition of arterial tension is due to loss of *vaso-motor* tone, producing a relaxed condition of the arterial and capillary systems and permitting a free outflow from the arteries. This also is the chief cause of the precipitous descent from the apex of the percussion wave. This interpretation is in accordance with the teachings of clinical experience. The sensation imparted by the pulse to the finger on the artery is that the blood courses along the artery in distinct waves to be completely emptied in the interval. Also the sense of coldness of the extremities, which the patient experiences as the case advances, and the purple hue of the fingers and toes, denotes a loss of *vaso-motor* tone, with a relaxed condition of the capillary system and diminished cardiac power. The advance of the purple hue up the extremities during *shiysohin* (and also frequently previous to and denoting the approach of *shiyoshin*), points also to continued loss of *vaso-motor* tone, with increased relaxation of the vascular system and accumulation of blood in the capillaries.

*The elements of danger in kakke are found in the impaired condition of the heart and circulation.* It will be noticed that the relation which exists between the condition of the vascular system and the general condition of the patient is subject to great variation. (*a*) The tracings in some cases show very irregular action of the heart, an abnormal high and sudden upstroke of the ventricular systole with loss of arterial tension, when the general symptoms were not those of greater danger. This is a very common condition; and cases are frequently met with where this contrast is so great that the patient is considered suffering from serious functional derangement or neurosis of the heart, while the ordinary symptoms of kakke are so slight as to be passed by unnoticed. (*b*) Again, when the general symptoms were grave, the tracings often show but slight deviation from the normal. (*c*) However grave the general symptoms may be, so long as the condition of the circulation and respiration remain good, there is no occasion for alarm. But however mild the general symptoms may be, if the condition of the circulation is much impaired, the indications are those of grave danger. The physician must look to the condition of the heart and circulation in kakke to determine the elements of danger in the case.

*The extent to which the heart and* VASO-MOTOR *system are affected in kakke, is relatively subject to considerable change.* The relaxed condition of the arterial and capillary system shows that the *vaso-motor* nerves of the sympathetic system are affected. (*a*) In some cases the sudden and tall upstroke of the percussion wave shows that the muscular grasp of the heart upon the contained blood is strong, giving a vigorous ventricular systole; while in the same tracing there is a precipitous fall from the apex of the percussion wave, a fully dicrotic or hyperdicrotic pulse with greatly reduced arterial tension, denoting a marked loss of *vaso-motor* tone. (*b*) Again, in other cases with weak cardiac action, there is a measure of arterial tension. (*c*) My experience with the sphygmograph in kakke has taught me that loss of *vaso-motor* tone is fraught with graver danger than loss of cardiac muscular power.

When there is marked dicrotism, loss of *vaso-motor* tone, and a relaxed arterial and capillary system with free outflow, even though the sudden and high upstroke of the percussion waves show favorable cardiac power, *shiyoshin* is liable to occur at any time.

In the above condition the toiling heart may at any time become exhausted, the powers of circulation suffer a partial collapse, and the phenomena of *shiyoshin* be developed.

*Addenda.*—Kakke presents some peculiar features of interest for study and investigation, among which we may mention:

*Age.*—Age as a predisposing cause to kakke is readily admitted by those who have had some experience with this disease. The most susceptible age is from sixteen to twenty-eight or thirty-two. A very large ratio of those seen in general practice will be young men between the ages of seventeen and twenty-five. I have never met with a case under twelve years of age, and from extensive inquiry, have not heard of a case under eleven. Children appear to en joy an absolute immunity from kakke. I have never met with a case over sixty-three years of age, and am informed that it very seldom occurs over sixty and never over sixty-five.

*Recurrence.*—One attack of kakke appears to render the patient more liable to subsequent attacks. A large number of those seen will state that they have suffered repeatedly from kakke in successive years. Many have had from three to five, seven, and ten attacks in as many successive years. Occasionally you will meet those who have repeatedly suffered from kakke, yet have one or two years' remission. Last year I

met with two persons, each of whom had suffered from kakke for the last twenty successive years.

*Sex.*—Women are much less likely to be affected with kakke than men. A common estimate among the Japanese for the number of cases of kakke occurring among women is 4–6 per centum. But this estimate is much too low. While women do enjoy a peculiar immunity from this disease, the difference between the number of women and men affected is not so great as would at first appear. Women generally have the disease in a much milder form than men; and many of them affected with kakke have it so light that they do not apply for medical aid. Of the number of cases recorded in my case-book, over 16 per cent. have been women; and an estimate of from 10 to 15 per cent. would be a close approach to the ratio of female cases occuring in kakke.

*Puerperal Kakke.*—Though women in general enjoy a marked immunity from kakke, yet in the puerperal state they are quite subject to this disease, and when taken in this condition it is attended with a marked fatality. Of the female kakke cases coming under my observation over 36 per cent. have been puerperal. Kakke may occur during lactation. In such cases it is not attended by special fatality; but it is very liable to occur shortly before or after child-birth, and in such cases it is most likely to be severe and attended with a high rate of mortality. Of such cases falling under my observation over 65 per cent. have been fatal.

*Urine.*—It is but seldom that traces of albumen are found in the urine of kakke patients. Of the cases coming under my observation, the urine of three or four contained for a short time a small quantity of albumen, that showed no traces of it after recovery. In almost all kakke cases the urinary secretion is diminished, and if the case is severe very markedly diminished. It frequently runs down to 900, 600, 300 cubic centimeters in twenty-four hours, and occasionally as low as 200 or 150 c.c. In some cases under my observation it has run down as low as 100–50 c.c. in twenty-four hours for several successive days, yet no œdema occurred. But such cases are most likely to die. The importance of keeping up the secretion of urine to the fullest extent should never be lost sight of in treating cases of kakke. If the circulation is not specially affected and the secretion of urine can be kept up to a reasonable quantity of 900 or 750 c.c. per day, the case may be considered hopeful. But if this secretion runs down below 600 or 500 c.c. in twenty-four hours, and can not be brought up, the case should be considered doubtful; for though the condition of the circulation be good, with so small a secretion of urine the powers of the circulation will soon show signs of failure. The secretion of urine frequently ceases some hours before death.

*Paralysis of the Bladder.*—Partial paralysis of the muscular walls of the bladder are occasionally met with in cases of kakke, so that micturition becomes tedious and imperfect. But if the case progresses favorably this will pass off after some days. A few cases of complete paralysis of the bladder, a short time before death, have also come under my observation; when, although the patient had not urinated for some time, yet a reasonable amount was drawn off with a catheter.

## Figures and Tables

**Figure f1:**